# Retraction: Cord blood stem cells inhibit epidermal growth factor receptor translocation to mitochondria in glioblastoma

**DOI:** 10.1371/journal.pone.0327905

**Published:** 2025-07-11

**Authors:** The PLOS One Editors

Following the publication of this article [1], concerns were raised about the results presented in Figs 1, 3, 4, and 7. Specifically,

- The following panels appear similar:
  - Fig 1D pFAK and Fig 7A pEGFR when flipped horizontally
  - Fig 1D GAPDH of this article [1], Fig 3G GAPDH of this article [1] when flipped vertically, Fig 4C GAPDH of this article [1], Fig 7G GAPDH of this article [1], and Fig 1B GAPDH (right panel) of [2, retracted in 3]
  - Fig 3E β-Actin of this article [1], Fig 5C β-Actin of [2, retracted in 3], and Fig 5F β-Actin of [4, retracted in 5]
  - Fig 3G FAK and Fig 7A pFAK
  - Fig 3G c-Src and Fig 7A p-c-Src
  - Fig 7C GAPDH of this article [1], Fig 5D GAPDH of [4, retracted in 5], Fig 5A GAPDH of [2, retracted in 3], Fig 5B GAPDH of [2, retracted in 3]
  - Fig 7E pEGFR and Fig 7I RAF-1
  - Fig 7G MEK1 and Fig 7G STAT3
  - Fig 7I STAT-3 of this article [1] and Fig 6B TNF-α of [4, retracted in 5] when flipped horizontally
- The following panels appear to partially overlap:
  - Fig 3I c-Src of this article [1], Fig 7C p-c-Src of this article [1], Fig 7I PI3K of this article [1], Fig 1B pAkt^Ser473^ (left panel, lanes 2-5) of [2, retracted in 3], and Fig 1B pAkt^Ser473^ (right panel, lanes 2-5) of [2, retracted in 3]

The authors did not respond to the journal’s communications.

In light of the above concerns that question the reliability and integrity of these results, the *PLOS One* Editors retract this article.

All authors either did not respond directly or could not be reached.

In addition to the above concerns, the PLOS Editors are also aware of similarities between results presented in Fig 3 and 7 of this article [1] and results presented in Figs 3 and 5 of [6]. PLOS notes that [6] was published later by researchers who do not appear to share affiliations with the authors of [1].
